# Research on the enhancement material and culture method of soil aggregates composed of feldspathic sandstone and sand

**DOI:** 10.1038/s41598-024-67073-y

**Published:** 2024-07-13

**Authors:** Juan Li, Jinbin Li, Dongwen Hua, Shaowei Li, Zhe Pang, Hongli Jiang

**Affiliations:** 1 Key Laboratory of Cultivated Land Quality Monitoring and Conservation, Ministry of Agriculture and Rural Affairs, Institute of Land Engineering and Technology, Shaanxi Provincial Land Engineering Construction Group Co., Ltd., Xi’an, 710021 China; 2 Key Laboratory of Degraded and Unused Land Consolidation Engineering, Ministry of Natural Resources, Shaanxi Provincial Land Engineering Construction Group, Xi’an, 710021 China

**Keywords:** Agglomerate, Material, Mean mass diameter, Geometric mean diameter, Fractal dimension, Biogeochemistry, Ecology

## Abstract

The Mu Us Sandy Land is a region characterized by wind-blown sand and soil erosion in northern China. To enhance the soil quality of this area, various organic materials were incorporated into the mixed soil at a volume ratio of 1:2 for feldspathic sandstone to sand. Culture was conducted in the field and under constant temperature conditions in laboratory culture chambers. Four treatments were established in the experiment, each calculated based on weight ratio and controlled (with no organic material added, CK); single application of straw (5% straw, P1); single application of biochar (5% biochar, P2); combined application of biochar and straw (5% biochar + 5% straw, P3). After 90 days of culture, soil samples were collected for analysis of various indicators such as soil aggregate particle size distribution, water stability of soil aggregates, mean weight diameter, mean geometric diameter, and fractal dimension using dry sieving and wet sieving methods. The objective is to establish a scientific basis and provide technical support for addressing the challenges associated with compound soil and implementing rational fertilization measures. The research results indicate that: (1) The quantity of aggregates > 0.25 mm under different treatments follows the order CK < P1 < P2 < P3, and the differences between treatments are significant (P < 0.05); (2) Soil water stability, mean weight diameter (MWD), mean geometric diameter (GMD), and fractal dimension of soil aggregates in compound soil with different organic material additions are superior to the control, and the effect of biochar on improving soil aggregates is better than that of corn straw. The combined application of both significantly improves the effect compared to single applications. In both culture modes, under wet sieving, the P3 treatment shows the highest MWD and GMD of soil aggregates, with an increase ranging from 3.45% to 85% and 4.55% to 38.46%, respectively, compared to other treatments. (3) The trend of fractal dimension among treatments is consistent: P3 < P2 < P1 < CK, and the differences between treatments are significant (P < 0.05). Moreover, there is a good negative correlation linear equation relationship between the fractal dimension (y) and WR > 0.25 (x) of compound soil, with a correlation coefficient of up to 0.9851. In conclusion, the incorporation of organic materials can effectively enhance the proportion of macroaggregates in compound soil consisting of Feldspathic sandstone and sand, thereby improving soil stability and erosion resistance. The optimal outcome is achieved through the combined application of biochar and straw. Indoor culture proves to be more effective than field culture, while wet sieving accurately reflects the structural characteristics of compound soil under both dry and wet sieving treatments.

## Introduction

Ecosystems play a crucial role in supporting human survival and development. Yet, the rise in population and industrial activities has caused the exploitation and depletion of Earth's ecosystems and resources. This has caused significant harm to vegetation, soil, and water bodies, resulting in the degradation of various ecosystems and sparking a range of ecological and environmental issues^1^. Sand ecosystems are known for their high productivity, substantial biomass, diverse biodiversity, and important social and economic contributions^2^. The Mu Us Sandy Land in China is distinguished by its favorable hydrothermal conditions, dense population, and vibrant economic activities^3^.

The Mu Us Sandy Land is known for its abundance of Feldspathic sandstone and sand, which are prone to weathering when exposed. This weathered sandstone easily turns into dust during windy conditions and is susceptible to erosion by rainwater, leading to severe soil erosion in the region^4^. According to relevant analysis, the Yellow River transports approximately 190 million tons of sand annually, with a majority being weathered material from feldspar sandstone^5^. The loose soil structure of Mu Us Sandy land leads to water and nutrient loss, making it challenging to create soil aggregates^6^. In windy weather, dust storms are common, and without proper protective measures, desertification can easily occur^4–6^. The ecological and environmental problems in the area are primarily caused by the abundance of Feldspathic sandstone and sand. Through scientific utilization, these materials can be mechanically synthesized and physically bonded to create a sandstone amalgam, which can then be used to form compound soil^7^. The compounded soil demonstrates exceptional sand-fixing, water-retaining, and agglomeration effects by leveraging the complementary characteristics and properties of its components. This improvement is primarily due to enhancing the unstable structures of both materials, ultimately increasing the stability of the soil mass.

Soil aggregates are considered the building blocks of soil composition and are commonly utilized to evaluate key aspects of soil structure. Numerous scholars, both domestic and international, have conducted thorough research on indicators of soil aggregate stability. Typically, research analysis can be carried out using two methods: water stability aggregates and force stability aggregates^8–10^. Indicators such as water stability aggregate stability rate, mean weight diameter, and mean geometric diameter are important for accurately reflecting soil stability^11–13^. Currently, research on soil aggregates mainly focuses on the dynamic changes influenced by anthropogenic factors such as fertilization, straw returning, and tillage^14–17^. The addition of straw and biochar to purple soil effectively improves its internal soil structure, with straw showing better improvement effects than biochar^18,19^. Research on sandy ginger black soil suggests that the combined application of straw and biochar yields the best improvement effects^20^. Straw returning with biochar significantly increases the content and stability of large aggregates in loamy clay soil and also enhances the content of large aggregates in red soil^19,20^. Studies analyzing the effects of straw and biochar application on soil aggregates in loess soil show that combining biochar with straw and fertilizer contributes most to the increase in organic carbon content of large aggregates^21,22^. It can be seen that there are certain differences in the application effects of straw and biochar, which may be limited by the carbonization temperature and straw particle size during biochar production^19–22^. For Mu Us Sandy Land, the application of straw and biochar is a relatively economical choice to improve soil fertility, and the application effect needs to be further studied. In this study, the research findings from Han Jichang's research team's extensive work were utilized^23^. When the volume ratio of pisha sandstone to sand is 1:2, the composite soil structure exhibits greater stability and leads to higher crop yields^23^. By adding different types and amounts of organic materials and utilizing both field and indoor culture modes, this study aims to investigate changes in soil aggregate indicators and correlations between various indicators, providing scientific support for the remediation of sandy soil and enhancing its stability. Therefore, there are two innovations in this study: firstly, the improvement effect of single organic material and mixed organic material on the stability of composite soil aggregates was proved; secondly, the influence of organic materials on the stability of compound soil aggregates under field culture and indoor culture was revealed.

## Materials and methods

### Experimental materials

**Figure 1:**
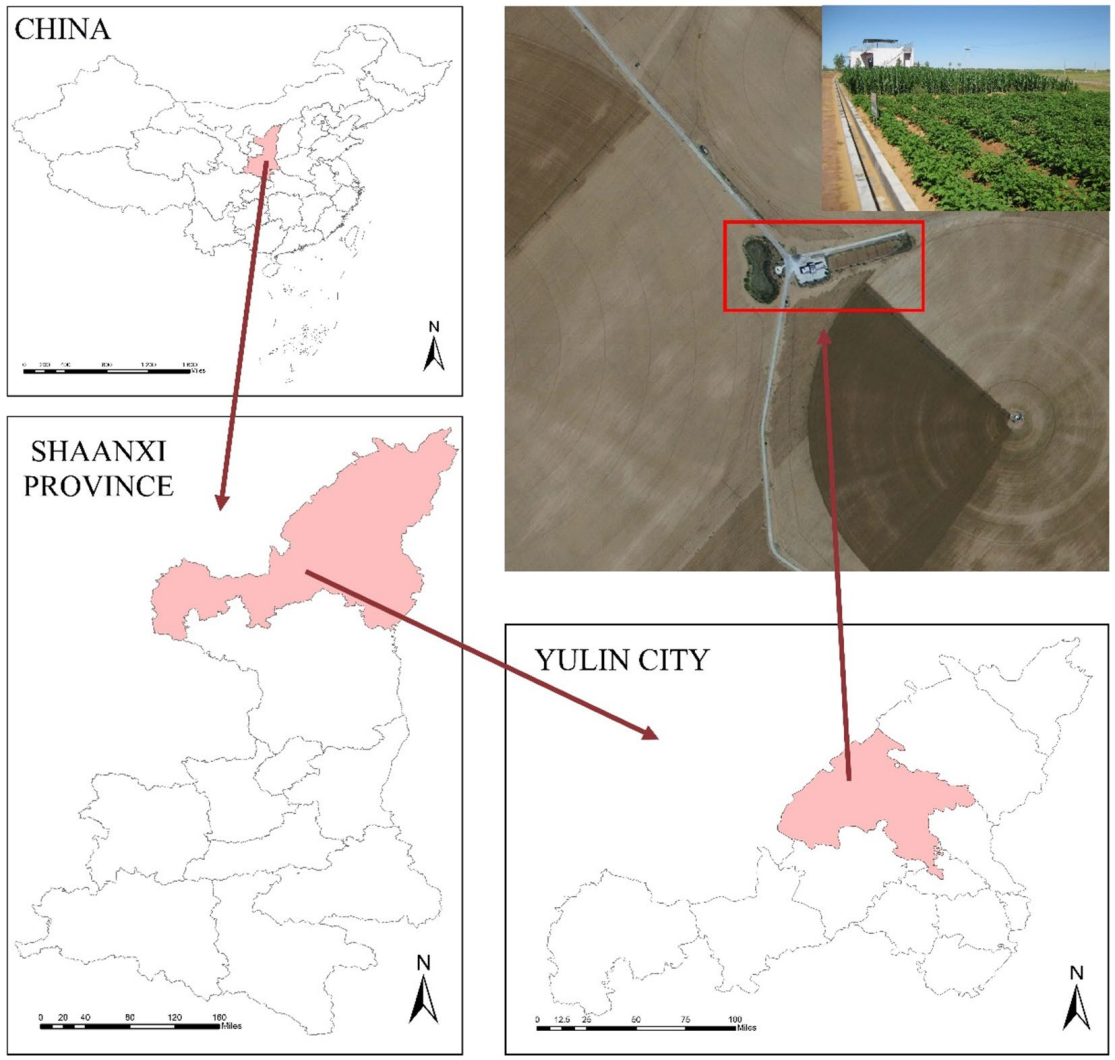
Location of the test site.

**Figure 2:**
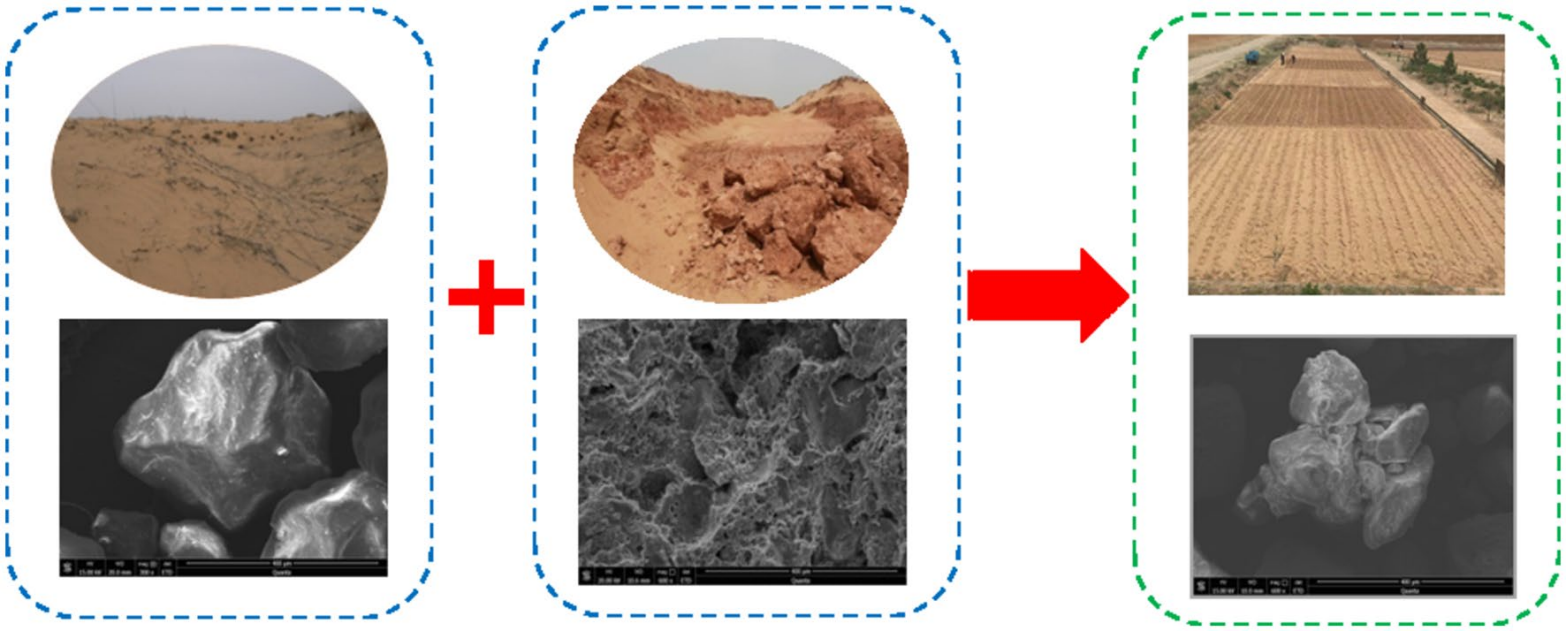
Sand, arsenic sandstone, and compounded soil images and SEM images.

The experimental Feldspathic sandstone and sand were collected from the experimental base of Yulin Field Scientific Observation and Research Station (Dajihan Village, Xiaojihan Township, Yuyang District, Yulin), as shown in Fig. 1. The feldspathic sandstone and sandweare staggered in the test area, and there was no obvious transport distance, which can solve the cost problem of loess transport. They were mixed in a volume ratio of 1:2 and determined to be sandy loam in texture, as depicted in Fig. 2. Analysis showed that the collected Feldspathic sandstone had low nutrient element content, with phosphorus, organic matter, and total nitrogen contents of 0.02%, 0.65%, and 0.75%, respectively.

### Biochar material preparation

The corn straw in this study was selected as raw material, washed six times to remove surface adhesion and dust, and initially dried, dried and crushed in an oven at 70 ~ 80 °C overnight, weighed 20 g corn straw in a crucible, covered, charred in a programmed temperature controlled Muffle furnace at 400 °C for 6 h, cooled to room temperature, and then removed. The carbonized products were treated with 200 mL 1 mol/L HCl solution for 12 h, the ash was removed, filtered, washed with distilled water until neutral, and dried at 70 ~ 80 °C overnight.

### Experimental design

The experiment was divided into indoor and field culture experiments, with four treatments established. The original Feldspathic sandstone to sand volume ratio was 1:2. The control group (CK) used composite soil without any organic materials. Organic materials, specifically corn straw and biological carbon, were added at a 5% volume ratio after being passed through a 1 mm screen (Table 1). An indoor culture experiment was conducted with test materials weighing 2500 g in each test pot, including soil samples and added materials. Five repetitions were set, with 70% distilled water of field water capacity added to each treated sample. The pots were completely sealed with plastic and had 5 reserved gas exchange ports arranged in a plum shape. Using a weighing method daily, the moisture lost by evaporation was replenished promptly to maintain soil moisture content within the field water capacity range. In the field culture experiment, conducted at the Qinling Field Monitoring Central Station in Meixian County, Baoji, Shaanxi Province, with a plot area of 25m^2^, each treatment was repeated three times. Biological carbon was applied before the experiment began, spread evenly on the surface, and manually cultivated into the 0–20 cm soil layer. Straw was applied through shallow rotation, with ploughing depths of 0-20 cm.

**Table 1:** Experimental design.

Treatments	CK	P1	P2	P3
Field study (field)	non-additive	5% (maize straw)	5% (biochar)	5% (biochar) 5%(maize straw)
Laboratory Study (24℃ incubator)	Non-additive	5% (maize straw)	5% (biochar)	5% (biochar) 5% (maize straw)

### Sample collection

The experiment commenced on March 1, 2023, and concluded on July 1, spanning a duration of 3 months. Following the experiment, soil samples from the pot were collected using the quartering method, with the weight of the samples representing half of the total weight. The soil was then fragmented along the natural cracks until reaching approximately 10 mm in size. Subsequently, the soil was air-dried naturally, sieved through screens with particle sizes of 5 mm and 2 mm, and a 200 g mixed sample was weighed based on the proportion of particle sizes.

### Determination indicators and methods

The particle size distribution and stability of soil aggregates are assessed through dry sieving and wet sieving methods^24–26^. Dry sieving involves separating soil samples into different particle size fractions using an oscillating sieve shaker to determine aggregate stability at each size grade. Wet sieving employs an aggregate analyzer to measure stability, with water-stable aggregates at each particle size being washed into aluminum containers using distilled water, followed by drying and weighing for mass calculation.

### Data analysis

Soil aggregate parameters. Typically, parameters such as the percentage content of soil aggregates > 0.25 mm (R_0.25_), water-stable aggregate stability rate (WASR), mean weight diameter (MWD), mean geometric diameter (GWD), and fractal dimension (D) are selected for calculation and explanation^27^. The calculation formulas are as follows:

1 $$W_{i} = \frac{{W_{di} /W_{wi} }}{200} \times 100\%$$

2 $$R_{0.25} = \sum\limits_{i = 1}^{n} {(W_{i} )}$$

3 $$WSAR = WR_{0.25} /DR_{0.25} \times 100\%$$

4 $$\text{MWD}=\sum_{i=1}^{n}(\overline{{x }_{i}}{w}_{i})$$

5 $$\text{GMD}=\text{Exp}\left[\frac{\sum_{i=1}^{n}{m}_{i}\text{ln}\overline{{x }_{i}}}{\sum_{i=1}^{n}{m}_{i}}\right]$$

6 $$\text{ln}\left[\frac{M(r<\overline{{x }_{i}})}{{M}_{T}}\right]=(3-D)\text{ln}\left(\frac{\overline{{x }_{i}}}{\overline{{x }_{max}}}\right)$$

$\overline{{x }_{i}}$ represents the mean diameter of the aggregate at the i-th particle size grade. W_i_ denotes the proportion of the weight of aggregates at the i-th particle size grade; W_i_ signifies the weight of aggregates at different particle size grades in the composite soil. DR_0.25_/WR_0.25_stands for the percentage content of > 0.25 mm force-stable/water-stable aggregates (%); M(r < $\overline{{x }_{i}}$) represents the weight of aggregates with a particle size smaller than r; that is, the maximum particle size of the aggregates.

Data analysis was conducted using Excel 2022 for initial processing, followed by statistical analysis of the data using Spss software. Multiple comparisons were performed using LSD (Least Significant Difference).

## Results and analysis

### Composite soil aggregates

**Figure 3:**
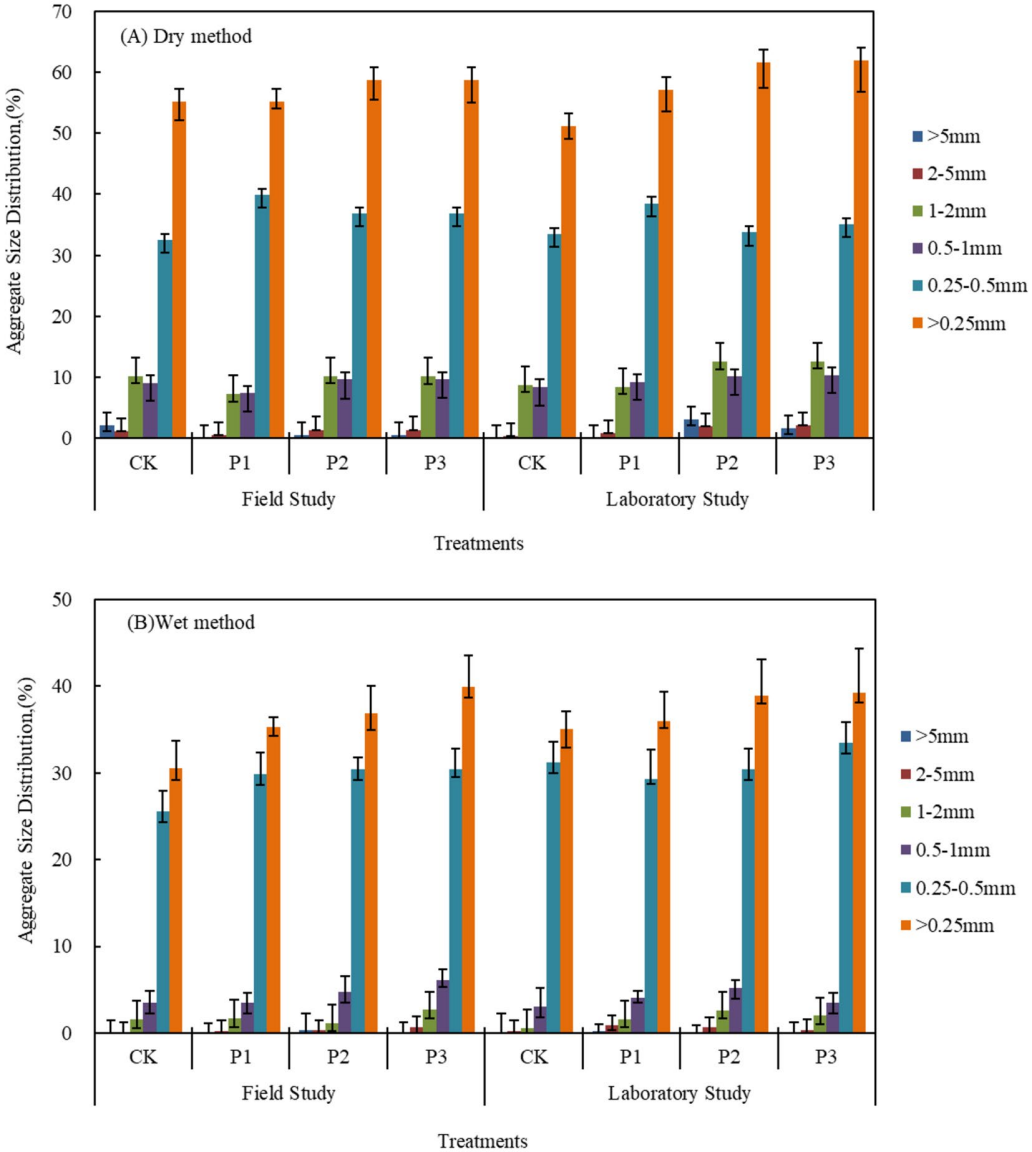
Percentage content of aggregates with different granule diameters under different additives was analyzed.

The impact of soil aggregate structure on soil fertility and stability is a topic of increasing research interest^28,29^. Soil aggregates play a crucial role in enhancing soil aeration, water retention, and nutrient adsorption. Different types of aggregates have varying abilities in these functions^30^. As illustrated in Fig. 3, both field and laboratory studies have shown that the composition of aggregates of varying particle sizes in composite soils with different additives exhibit a consistent trend under dry and wet sieving treatments. Specifically, the content of aggregates larger than 0.25 mm follows the order: CK < P1 < P2 < P3, with statistically significant differences observed between the treatments (P < 0.05). Under the field culture treatment P3, there is a higher content of aggregates > 0.25 mm observed under both dry and wet sieving treatments when compared to other treatments. Specifically, the content is higher by 16.98%, 17.01%, 10.08% and 21.05%, 8.45%, 2.15%, respectively. Similarly, under the experimental culture, the content is higher by 30.36%, 12.83%, 8.19% and 11.98%, 9.12%, 0.82%, respectively. A comprehensive analysis suggests that the addition of straw and biochar promotes the formation of soil aggregate structure, leading to a significant increase in the formation of large aggregates.

### Water stability of aggregates

**Figure 4:**
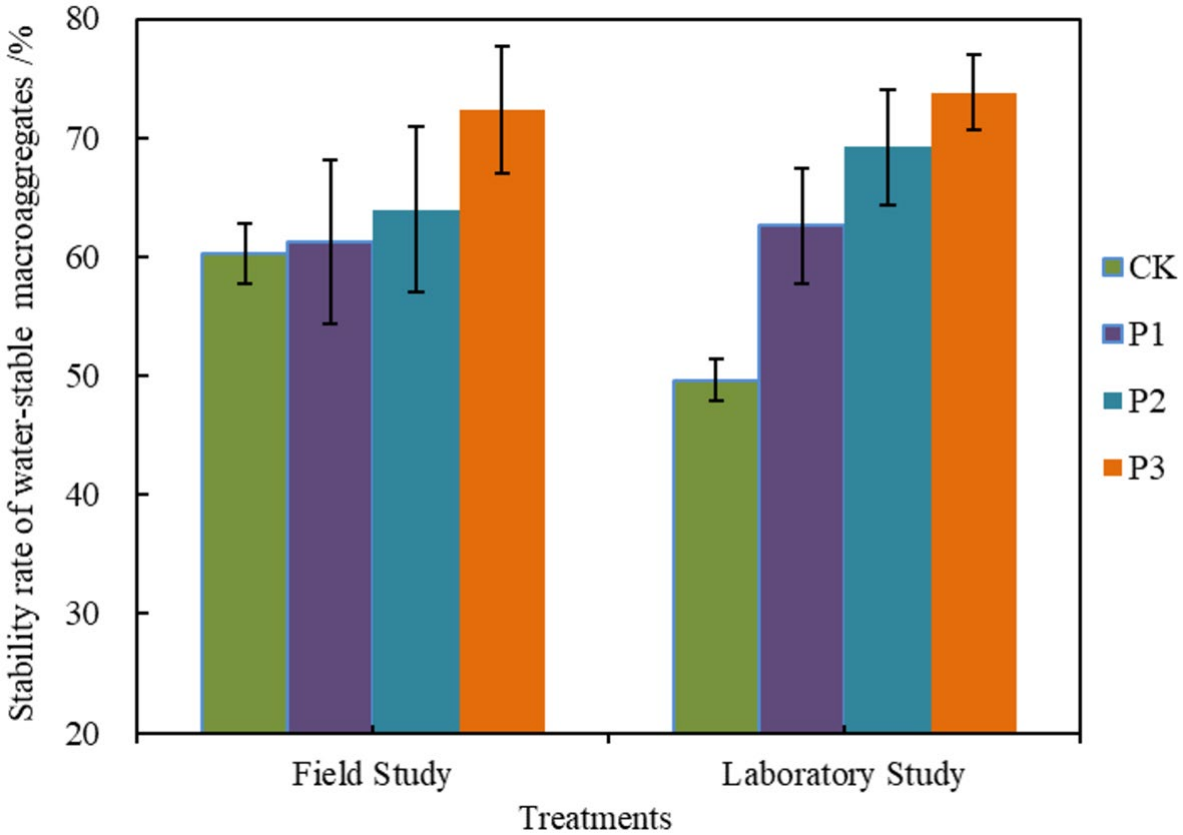
Effect of different Organic material type and adding amount on the stability of composite soil.

The impact of various organic materials and their quantities on the water stability of composite soil aggregates is shown to vary (Fig. 4), with significant differences observed between treatments (P < 0.05). Across different cultivation methods, a consistent trend is observed in the water stability of aggregates with a content > 0.25 mm, where the sequence from lowest to highest is CK < P1 < P2 < P3, with significant differences between treatments (P < 0.05). In both field and culture experiments, the water stability of aggregates under treatments P1, P2, and P3 is higher compared to the control CK. The percentages range from 1.52%, 6.07%, and 19.95% to 26.13%, 39.37%, and 48.64%, respectively. Treatment P3 demonstrates the highest stability, showing an increase ranging from 13.09% to 19.95% and 6.65% to 48.64% when compared to other treatments. A comparative analysis indicates that the use of materials can enhance the stability of composite soil, with biochar application showing superior results to straw application alone. Moreover, when combined, the stability of composite soil is notably enhanced.

**Figure 5:**
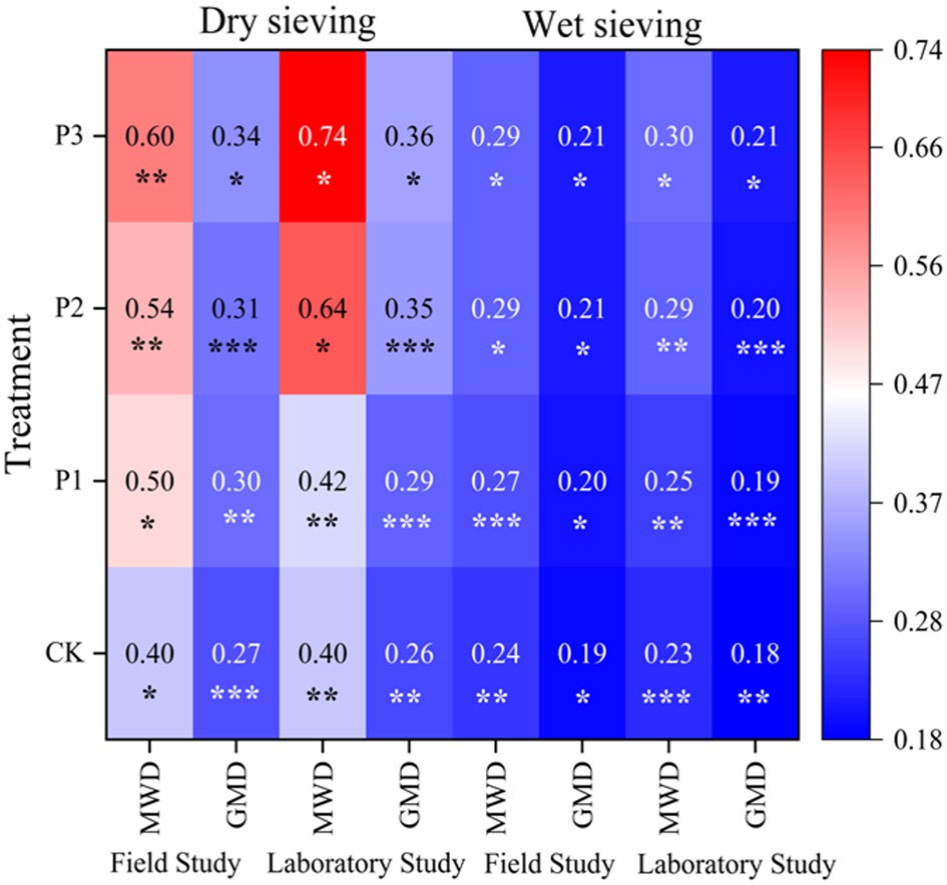
Effect of different organic material types and amount on the stability parameters of the compound soil. * indicated significant difference between treatments (P < 0.05); ** indicated significant difference between treatments (P < 0.01). The same below.

Soil aggregate mean weight diameter (MWD) and mean geometric diameter (GMD) are among the evaluation indicators of soil structure^25,26^. These parameters primarily reflect the size and distribution arrangement of soil aggregates. Higher values of MWD and GMD indicate a more significant soil aggregation effect, suggesting stability and maturity in development stages. They also play a role in promoting soil water retention and fertility to some extent^31^. Under dry and wet sieving methods, the MWD and GMD of composite soils under different treatments are shown in Fig. 5. The performance of soil stability indicators under different treatments in dry and wet sieving methods is consistent with that of water stability of aggregates. The results show that the effects of organic material addition are better than untreated controls, and the improvement effects of composite organic materials on composite soil are better than single organic materials. Under both field and laboratory culture experiments, treatment P3 exhibits optimal performance in MWD and GMD under dry and wet sieving methods, with no significant differences observed compared to the control CK treatment (P < 0.05). In field and laboratory culture experiments, when comparing treatment P3 to other treatments using dry and wet sieving methods, the increase in MWD and GMD of composite soil ranges from 11.11% to 50.00%, 9.68% to 25.93%, 3.57% to 20.83%, 4.76% to 15.79%, and 15.63% to 85.00%, 12.50% to 38.46%, 3.45% to 30.43%, 4.55% to 27.78%, respectively. Comparative analysis under different culture conditions shows that in laboratory culture, the MWD and GMD of composite soil treated with P3 are better than those in field culture experiments using dry and wet sieving methods. There were increases of 23.33%, 5.88%, 3.45%, and 4.55%, respectively, with no significant differences between treatments (P < 0.05).

### Fractal dimension

**Figure 6:**
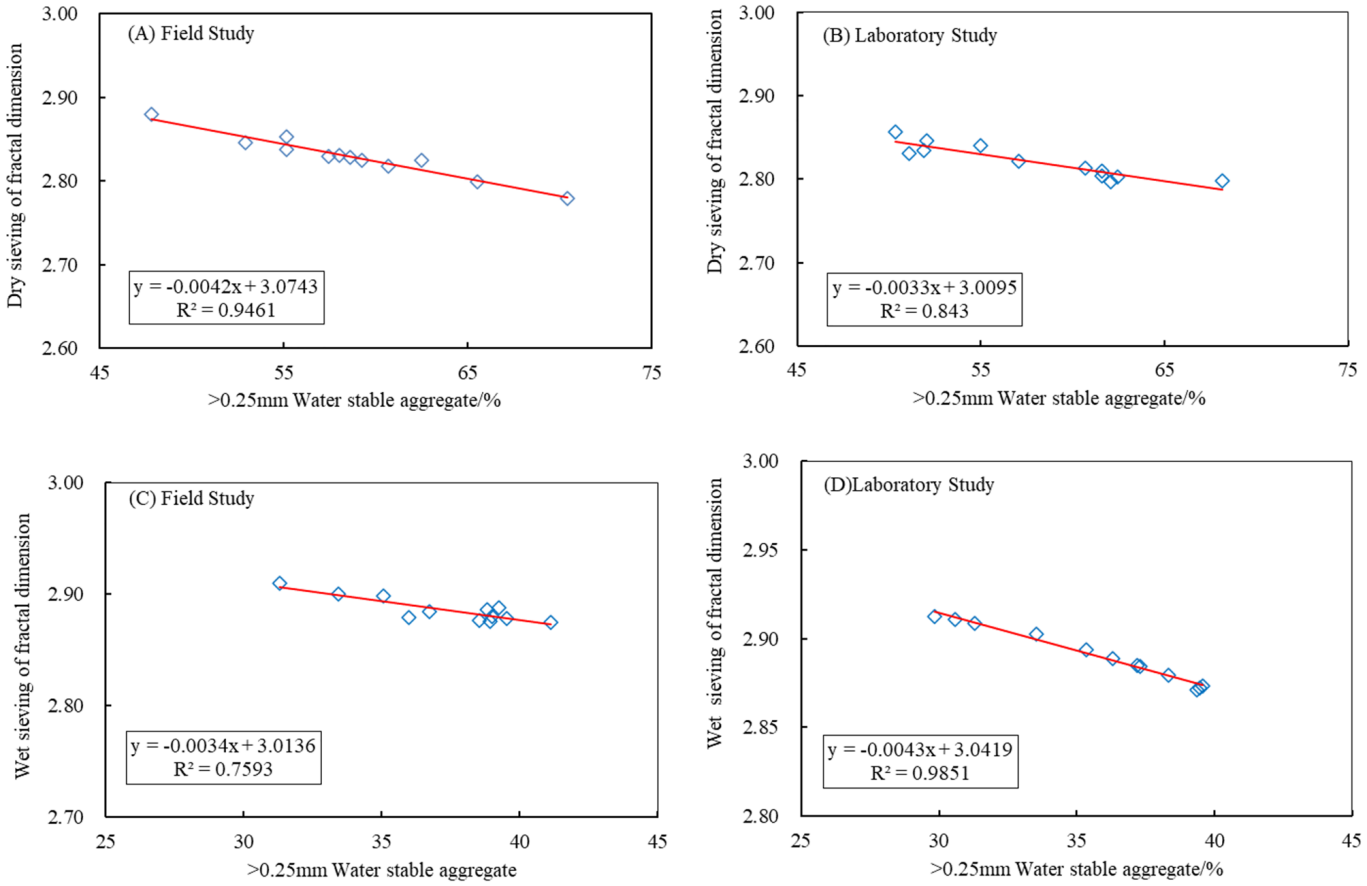
Correlation of Fractal dimension with > 0.25 mm soil aggregates.

The fractal dimension is a more precise indicator that reflects the characteristics of soil aggregates. By using the equation for determining soil particle size-weight distribution, the fractal equation for particle size distribution can be directly calculated, enabling the accurate and direct computation of soil particle fractal dimensions. This measurement primarily captures variations in soil particle sizes and, to some extent, the level of uniformity in texture distribution. In general, a higher fractal dimension indicates lower stability, and conversely^32,33^. The fractal dimension of soil is negatively correlated with the stability of soil aggregates (Table 2). The influence of different additives and their quantities on the fractal dimension of composite soil is shown in Fig. 6. Under dry sieving, the fractal dimension distribution ranges from 2.79 to 2.85 for field and experimental cultures, while under wet sieving, the range is 2.85 to 2.90 and 2.85 to 2.91, respectively. The trend of fractal dimension across treatments shows P3 < P2 < P1 < CK consistently, with significant differences between treatments (P < 0.05). Treatment P3 demonstrates the most notable enhancement in the composite soil structure.

**Table 2:** Effect of different organic material types and amount on the fractal dimension of composite soil.

Treatment	Dry sieving		Wet sieving	
	Field study	Laboratory study	Field study	Laboratory study
CK	2.85***	2.86**	2.90***	2.91**
P1	2.84**	2.85**	2.88***	2.89**
P2	2.83**	2.82***	2.87**	2.88**
P3	2.79 *	2.80*	2.85*	2.85*

*indicates a significant difference at 5% level, **indicates a significant difference at 1% level, and ***indicates a significant difference at 1% level.

### Relationship analysis

A higher percentage of aggregates > 0.25 mm is advantageous for creating a well-aggregated structure and enhancing the soil's ability to withstand external forces. The relationship between the fractal dimension of composite soil and the percentage content of aggregates > 0.25 mm under different treatments is shown in Fig. 5. Analysis demonstrates a strong linear relationship and a notable negative correlation between the fractal dimension of composite soil aggregates and the percentage content of aggregates > 0.25 mm. Under dry sieving, the relationship between the fractal dimension (y) and WR > 0.25 (x) under field and experimental culture conditions is y = -0.0042x + 3.0743 (R^2^ = 0.9461) and y = -0.0033x + 3.0095 (R^2^ = 0.843), respectively. Under wet sieving, the relationships are y = -0.0034x + 3.0136 (R^2^ = 0.7593) and y = -0.0043x + 3.0419 (R^2^ = 0.9851), respectively. The regression equations suggest that as the content of aggregates > 0.25 mm increases in composite soil, the soil structure and aggregation become more stable, resulting in enhanced erosion resistance. Conversely, a lower fractal dimension indicates a more mature stage of soil development.

## Discussion

### Effects of different materials on the composition of composite soil aggregates

Soil aggregates are discontinuous structural units formed by natural or anthropogenic processes, including clods, aggregates, and other units. Several studies have demonstrated that various organic materials, like biochar and straw incorporation, can significantly enhance soil organic carbon content and encourage the development of larger soil aggregates^10,11^. Biochar typically has a high specific surface area and contains numerous organic molecules, both of which can effectively facilitate the formation of large aggregates^34^. Long-term field experiments on straw incorporation into loess soil have indicated no significant improvement in soil aggregate composition, but there is an observed increasing trend in the content of large aggregates in the soil^35^. The analysis of Haplic Luvisols with straw and biochar application reveals that organic materials can greatly enhance the presence of large aggregates in the soil. However, the increase in advantageous particle size differs depending on the type of material added. For instance, straw incorporation significantly increases the content of aggregates > 1 mm, while the combined use of biochar and straw is conducive to the formation of aggregates > 0.25 mm^36^. This is consistent with the results of this study, where the content of aggregates > 0.25 mm follows the sequence CK < P1 < P2 < P3, and the differences between treatments are significant (P < 0.05). The addition of organic materials is advantageous for enhancing the structure of composite soil, with biochar demonstrating a greater impact compared to straw. This is primarily due to biochar's extensive specific surface area and substantial negative charge on its surface, which enhances the exchange capacity of soil cations and facilitates the formation of larger soil aggregates more efficiently than straw. However, the optimal effect is achieved through the combined application of both materials^37^. Specifically, under field culture, treatment P3 shows higher content of aggregates > 0.25 mm under dry and wet sieving treatments compared to other treatments by 16.98%, 17.01%, 10.08% and 21.05%, 8.45%, 2.15%, respectively; under experimental culture, it is higher by 30.36%, 12.83%, 8.19% and 11.98%, 9.12%, 0.82%, respectively.

### Effects of different materials on the stability and size of composite soil aggregates

The WASR of composite soil is a reliable indicator of soil structure stability, where a higher value signifies greater stability. Meanwhile, the MWD and GMD of soil aggregates can provide insights into the particle size distribution characteristics of aggregates following various tillage treatments. MWD and GMD are commonly utilized as metrics to assess soil aggregate condition, with larger values suggesting improved aggregation and enhanced stability of soil aggregates^37^.

Under various cultural practices, the WASR of composite soil displays a consistent pattern across different treatments, aligning with the distribution of aggregates > 0.25 mm in size, following the sequence CK < P1 < P2 < P3. The observed differences between treatments are statistically significant (P < 0.05). Previous research has indicated that the incorporation of wheat straw ash biochar into red soil resulted in a 28% increase in MWD of the soil, while the addition of herbaceous plant biochar to clay soil led to a 21% increase in MWD of soil aggregates^38,39^. The study found that both MWD and GMD exhibited a sequence of CK < P1 < P2 < P3 under different treatments. When dry sieving was used, the MWD and GMD increased by 25.00% to 50.00%, 11.11% to 25.93%, and 50.00% to 85.00%, 11.54% to 38.46% under field and laboratory conditions for each treatment compared to the control CK. Similarly, under wet sieving, the increases ranged from 12.50% to 20.83%, 5.26% to 15.79%, and 8.69% to 30.43%, 5.56% to 27.78%, respectively. The differences between treatments were found to be statistically significant (P < 0.05). Under both culture conditions, treatment P3 exhibits the highest MWD and GMD of aggregates, with increases compared to other treatments ranging from 3.45% ~ 85.00% and 4.55% ~ 38.46%, respectively.

### Fractal dimension of composite soil and its correlation analysis

Fractal dimension is a crucial indicator for quantitatively assessing soil structure stability. It can reflect the uniformity of texture in aggregates and provide insights into properties such as soil water, nutrients, air, and temperature. The expression of fractal dimension is meaningful and functional, enabling a comprehensive and rational representation of soil properties and functions^40^. Studies on straw incorporation have shown that the fractal dimension can indicate aggregate distribution and respond to changes in soil aggregates at varying straw incorporation rates. The fractal dimension is inversely related to the mean weight diameter and geometric mean diameter, suggesting that higher fractal dimension values are associated with less stable soil structures, while lower values are linked to more stable structures^41,42^. The results of this study reveal a consistent trend in the fractal dimension across treatments, with the order P3 < P2 < P1 < CK, and statistically significant differences between treatments (P < 0.05). Additionally, a strong negative linear correlation is observed between the fractal dimension of composite soil (y) and the content of aggregates > 0.25 mm (x), with a high correlation coefficient of 0.9851.

## Conclusion

1. A comprehensive analysis of soil aggregate stability parameters, such as the content of aggregates > 0.25 mm, mean weight diameter (MWD), geometric mean diameter (GMD), fractal dimension (D), and water-stable aggregate stability rate under dry sieving and wet sieving methods, revealed that the incorporation of organic materials enhances soil stability. This enhancement is evidenced by the significant increase in large soil aggregates, leading to an overall improvement and enhancement of soil structure.
2. Analysis of soil aggregate stability parameters under the addition of single organic material and mixed organic material, the improvement achieved with mixed materials is superior to that with single material, and the improvement effect is significant.
3. The impact of field culture and laboratory culture on the stability of composite soil with varying organic material additions was analyzed. Both culture methods were found to enhance the structure and stability of composite soil, yielding positive outcomes. Laboratory culture demonstrated better results than field culture. When comparing the characteristics of composite soil aggregates under dry sieving and wet sieving treatments, it was observed that wet sieving resulted in smaller data variability, providing a more accurate reflection of the structural characteristics of composite soil.

The structural stability of aeolian sand soil can be enhanced by incorporating biological straw and biochar. Subsequent experimental studies will focus on refining the structural properties of aeolian sand soil, with a particular emphasis on identifying the most effective material type and dosage. By systematically varying the type and quantity of added materials, this research aims to establish a sound scientific foundation for enhancing the structural integrity and overall quality of sandy land.

## Data Availability

All data generated or analyzed during this study are included in this published article.

## References

[1] Chen YQ, Li XB, Tian YJ, Tan MH (2009). Structural change of agricultural land use intensity and its regional disparity in China. J. Geogr. Sci..

[2] Kong DZ (1996). The ecological economic principle for comprehensive development and mangement of Maowusu Sandland. Pratacult. Sci..

[3] Zheng YRZ, Shi X (1998). The diagnosis and optimal design of high efficient ecological economy system in Maowusu Sandy Land. Chin. J. Plant Ecol..

[4] Wang RG, Wu XX (2009). New pattern to control Mu Us Sandland. Res. Soil Water Conserv..

[5] Yang F, Bi C, Cao M (2014). Simulation of sediment retention effects of the double seabuckthorn plant flexible dams in the Pisha Sandstone area of China. Ecol. Eng..

[6] Wang YC, Tun YG, Kou Q, Min DA, Chang YZ, Zhang RJ (2007). Definition of arsenic rock zone borderline and its classification. Sci. Soil Water Conserv..

[7] Wu Y, Yang ZF, Liu H, Cai HS, Wei M (2016). Effect of composition on nutrient of Pisha Sandstone. Yellow River.

[8] Qi YC, Wang YQ, Jun LM, Yu XS, Zhou CJ (2011). Comparative study on composition of soil aggregates with different land use patterns and several kinds of soil aggregate stability index. Trans. Chin. Soc. Agric. Eng..

[9] Six J, Elliott ET, Paustian K (2000). Soil structure and soil organic matter: II. A normalized stability index and the effect of mineralogy. Soil Sci. Soc. Am. J..

[10] Six J, Bossuyt H, DeGryze S, Denef K (2004). A history of research on the link between (micro)aggregates, soil biota, and soil organic matter dynamics. Soil Tillage Res..

[11] Dong JX (2021). Improving farmland soil physical properties by rotary tillage combined with high amount of granulated straw. Sci. Agric. Sin..

[12] Cong P (2019). Increasing straw incorporation rates improves subsoil fertility and crop yield in the Huang-Huai-Hai plain of China. Arch. Agron. Soil Sci..

[13] Zhu QL (2017). Effects of straw and waste application on soil aggregates and soil carbon, nitrogen and phosphorus in the Jasmine garden. J. Soil Water Conserv..

[14] Yuan JJ, Tong YA, Lu SH, Yuan GJ (2018). Biochar and nitrogen amendments improving soil aggregate structure and jujube yields. Trans. Chin. Soc. Agric. Eng..

[15] Samoraj M (2022). Biochar in environmental friendly fertilizers: Prospects of development products and technologies. Chemosphere.

[16] Baranian Kabir E, Bashari H, Mosaddeghi MR, Bassiri M (2017). Soil aggregate stability and organic matter as affected by land use change in central Iran. Arch. Agron. Soil Sci..

[17] Zhu GY, Shangguan ZP, Deng L (2021). Variations in soil aggregate stability due to land use changes from agricultural land on the Loess Plateau, China. CATENA.

[18] Lin HY, Zhou MH, Zhang BW, Li ZY, Zhu B (2020). Effect of long-term application of biochar and straw on soil organic carbon in purple soil aggregates of sloping uplands. Chin. J. Eco-Agric..

[19] Ghorbani M, Asadi H, Abrishamkesh S (2019). Effects of rice husk biochar on selected soil properties and nitrate leaching in loamy sand and clay soil. Inter. Soil Water Conserv. Res..

[20] Hou XN (2015). Effects of biochar and straw additions on lime concretion black soil aggregate composition and organic carbon distribution. Sci. Agric. Sin..

[21] Sailike A (2023). Restoration of grassland soil aggregate composition and total nitrogen distribution characteristics in Loess hilly area. Acta Ecol. Sin..

[22] Hu L, She DL, Yang Z (2022). Stability of soil aggregates and its differentiation characteristics in small watersheds in Loess hilly region of Northwestern Shanxi. Res. Soil Water Conserv..

[23] Han JC, Liu YS, Luo LT (2012). Research on the core technology of remixing soil by soft rock and sand in the Maowusu sand land region. China Land Sci..

[24] Pi HW, Huggins DR, Sharratt B (2019). Dry aggregate stability of soils influenced by crop rotation, soil amendment, and tillage in the Columbia Plateau. Aeol. Res..

[25] Lu JW, Li ZB (2002). Advance in soil aggregate study. Reach Soil Water Conserv..

[26] Lu RK (1999). Method of Analysis in Soil and Agro Chemistry.

[27] Yang PL, Luo YP, Shi YC (1993). Use the weight­size distribution tocharacterize the soil fractal features. Chin. Sci. Bull..

[28] Hansen V (2016). The effect of straw and wood gasification biochar on carbon sequestration, selected soil fertility indicators and functional groups in soil: An incubation study. Geoderma.

[29] Amezketa E (1999). Soil aggregate stability: A review. J. Sustain. Agric..

[30] Liu Y (2018). Soil Aggregates as affected by wetting-drying cycle: A review. Soil.

[31] Jozefaciuk G, Czachor H (2014). Impact of organic matter, iron oxides, alumina, silica and drying on mechanical and water stability of artificial soil aggregates. A ssessment of new method to study water stability. Geoderma.

[32] Zhang S (2021). Organic carbon, total nitrogen, and microbial community distributions within aggregates of calcareous soil treated with biochar. Agric. Ecosyst. Environ..

[33] Almajmaie A, Hardie M, Acuna T, Birch C (2017). Evaluation of methods for determining soil aggregate stability. Soil Tillage Res..

[34] Islam MU, Jiang FH, Guo ZC, Peng XH (2021). Does biochar application improve soil aggregation? A meta-analysis. Soil Tillage Res..

[35] Wang, Y. *Effects of Tillage Practices on Soil and Water Stability of Lou Soil Aggregate and Its Organic Carbon Distribution and Properties* (Northwest A & F University, 2012).

[36] Qiao DD (2018). Effect of boichar and straw with chemical fertilizers on soil aggregate distribution and organic carbon content in yellow cinnamon soil. Soil Fertil. Sci. China.

[37] Zhou HLYZ, Yang ZC, Li BG (2007). Effects of conservation tillage on soil aggregates in Huabei plain, China. Sci. Agric. Sin..

[38] Li J (2016). Effects of biochar application on soil organic carbon distribution and soil aggregate composition of red soils in Yunnan tobacco planting area. Huanjing Kexue Xuebao/Acta Scientiae Circumstantiae.

[39] Sun FF, Lu SG (2014). Biochars improve aggregate stability, water retention, and pore-space properties of clayey soil. J. Plant Nutr. Soil Sci..

[40] Gao MH (2020). Effects of straw and biochar returning on soil aggregates distribution and organic carbon content in brown soil. J. Plant Nutr. Fertil..

[41] Wang L, Li J, Li J, Bai WX (2014). Effects of tillage rotation and fertilization on soil aggregates and organic carbon content in corn field in Weibei Highland. Ying Yong Sheng Tai Xue Bao.

[42] Cheng K, Li J, Mao HL (2013). Effects of different rotational tillage patterns on soil physical properties in rainfed wheat fields of the Loess Plateau. Sci. Agric. Sin..

